# T-cell therapy against cancer mutations

**DOI:** 10.18632/oncotarget.2234

**Published:** 2014-07-19

**Authors:** Eric Tran, Steven A. Rosenberg

**Affiliations:** Surgery Branch, National Cancer Institute, National Institutes of Health, Bethesda, MD, USA

The ultimate goal of cancer therapy is to eliminate all tumor cells while sparing normal tissues. Although systemic chemotherapies can prolong survival in some patients with metastatic solid cancers, these responses are often short-lived and at the expense of significant toxicities to normal tissues because the molecules targeted by chemotherapeutic agents are not unique to the tumor. The paucity of truly unique tumor molecules that are targetable remains the major barrier toward developing effective treatments for patients with metastatic solid cancers.

All cancers contain somatic genetic alterations (mutations) which are truly unique to the cancer, and thus therapies that can specifically target mutations may provide therapeutic benefit in the absence of normal tissue toxicities. A hallmark of the human immune system is its ability to target an antigen with exquisite specificity and sensitivity. T cells are capable of mediating potent destruction of cells and tissues, both normal and malignant, provided they express the antigen that is targeted by the T cells. Consequently, T-cell based immunotherapies can mediate durable complete tumor responses in patients with metastatic melanoma and accumulating evidence indicates that these responses are mediated by T cells that target patient-specific mutations expressed by the patients' tumors [1-5]. The presence of mutation-reactive T cells in melanomas may be due to the high number of mutations, often hundreds to even thousands, found in these cancers which may increase the likelihood that a given mutation may be immunogenic. Like melanoma, smoking induced lung cancers also have a very high number of mutations and thus it is tempting to speculate that the clinical efficacy seen with anti-PD-1 immunotherapy in some lung cancer and melanoma patients [6] is attributable to mutation-reactive T cells. Given that most other solid tumors on average have fewer mutations, it is possible that mutation-reactive T cells may be absent or rare in the majority of solid tumors which would limit the therapeutic potential of targeting mutations with T cells for these cancers. However, in an exemplary case of a patient with progressing metastatic epithelial (cholangiocarcinoma) cancer with a relatively small number of mutations, 26, we found tumor-infiltrating CD4+ T cells that recognized a mutation in ERBB2IP specifically expressed by the patient's tumors [7]. The infusion of a highly pure population of these mutation-specific CD4+ T cells resulted in regression of all target lesions [7] which is ongoing as of the last follow-up at 8 months. Notably, all the aforementioned clinical responses occurred in the absence of normal tissue toxicity.

Together, these observations point to patient-specific mutations as ideal targets for immune-based therapies and we provide a blueprint for harnessing the immune system against mutations [7]. First, next-generation sequencing (whole-exome, whole-genome, and/or whole transcriptome) can be used to identify all genetic alterations expressed by the tumor. Second, minigenes or long peptides containing the mutation flanked by endogenous sequences are constructed and introduced into autologous antigen presenting cells (APCs), thereby allowing for the expression and presentation of the tumor mutations on any of the patient's MHC molecules. Third, T cells derived from tumor or peripheral blood are cocultured with the APCs expressing the mutations, and reactive T cells are identified and selected using standard immunological techniques. The mutation-reactive T cells are then further expanded and used for treatment. Alternatively, the genes encoding the mutation-reactive T-cell receptors (TCRs) can be isolated, introduced into autologous peripheral blood T cells, and used for therapy. One advantage of using mutation-reactive TCR-gene engineered cells is that the TCR can be introduced into less differentiated “younger” T-cell populations which mediate superior anti-tumor immunity compared to more differentiated “older” T cells in many preclinical tumor models.

Moving forward, critical questions and hurdles remain. Although all cancers contain genetic alterations, not all of these mutations are immunogenic. Do a minority or majority of patients with common solid tumors mount a T-cell response to at least one of their mutations? Our unpublished preliminary data in patients with metastatic gastrointestinal cancers suggests that the ability of the immune system to mount T-cell responses against their mutations may not be a rare event. But even if this observation holds true, the presence of mutation-reactive T cells infiltrating tumors that are progressing suggests that immunosuppressive/immunoevasive mechanisms are actively impairing these T cells, and thus strategies to overcome immunosuppression/immunoevasion may be required to enhance the efficacy of the mutation-reactive T cells. There are also practical hurdles to consider. Is it possible to treat large numbers of patients with this type of therapy? Since the tumor mutations and immune system are unique to every patient, the described treatment is highly personalized and relatively complex, requiring extensive laboratory expertise, manpower, and time (several months). However, if targeting mutations with T cells proves effective against common metastatic solid cancers, then it seems well within our reach to streamline the process and develop the infrastructure required to treat patients on a larger scale.
